# Understanding the molecular association between hyperkalemia and lung squamous cell carcinomas

**DOI:** 10.1186/s12881-020-01099-7

**Published:** 2020-10-22

**Authors:** Xianping Meng, Hongyan Lu, Xia Jiang, Bin Huang, Song Wu, Guiping Yu, Hongbao Cao

**Affiliations:** 1 Department of Radiology, Jiangyin People’s Hospital, Jiangyin, 214400 Jiangsu Province China; 2 Department of Cardiothoracic Surgery, The affiliated Jiangyin Hospital of Southeast University Medical College, Jiangyin, 214400 Jiangsu China; 3 Department of Psychiatry, First Hospital/First Clinical Medical College of Shanxi Medical University, Taiyuan, 030001 Shanxi Province China; 4 Department of Genomics Research, RD Solutions, Elsevier Inc, Rockville, MD 20852 USA

**Keywords:** Hyperkalemia, Lung squamous cell carcinomas, Mega-analysis, Pathway analysis, Multiple linear regression analysis

## Abstract

**Background:**

Previous studies indicated a strong association between hyperkalemia and lung squamous cell carcinomas (LSCC). However, the underlying mechanism is not fully understood so far.

**Methods:**

Literature-based data mining was conducted to identify genes, molecule, and cell processes linked to both hyperkalemia and LSCC. Pathway analysis was performed to explore the interactive network, common-target network, and common-regulator network for both disorders. Then, a mega-analysis using 11 independent LSCC RNA expression datasets (358 LSCCs and 278 healthy controls) was performed to test the hypothesis that genes influencing hyperkalemia may also play roles in LSCC.

**Results:**

There was a significant overlap between the genes implicated with both diseases (20 genes, *p*-value = 4.98e-15), which counts for 16% of all hyperkalemia genes (125 genes). Network analysis identified 12 molecules as common targets for hyperkalemia and LSCC, and 19 molecules as common regulators. Moreover, 19 molecules were identified within an interactive network, through which hyperkalemia and LSCC could exert influence on each other. In addition, meta-analysis identified one hyperkalemia promoter, *SPP1,* as a novel contributor for LSCC (LFC = 2.64; *p*-value = 2.81e-6). MLR analysis suggests geographical region as an influential factor for the expression levels of *SPP1* in LSCC patients (*p* value = 0.036, 0.054).

**Conclusion:**

Our results showed that there was a common molecular basis for the pathology of both hyperkalemia and LSCC, and that genes promoting hyperkalemia might also play roles in the development of LSCC. However, this study did not suggest hypercalcemia as a casual factor for LSCC.

## Background

Hyperkalemia is an elevated level of potassium (K+) in the blood (> 5.5 mmol/L), which is caused by abnormalities in the normal bone formation and degradation cycle, and leading to increased calcium (Ca2+) level in the blood serum [1]. About 30% of cancer patients demonstrate hyperkalemia [1, 2], especially in squamous cell carcinomas of head and neck, esophageal, cervical, and lung [2].

A strong association was reported between hyperkalemia and lung squamous cell carcinomas (LSCC) [3]. It is affecting patients in progressive stages, shortening survival times, and leading to poor prognosis [3]. Genetic mutations have also been reported to support the relationship between both diseases. Nielsen et al. found that ectopic *PTH*-producing squamous cell lung carcinoma associated with humoral hyperkalemia of malignancy [4]. Lorch et al. reported that EGFR-induced Ras-mitogen-activated protein kinase signaling accounts for high levels of PTHrP expression, and contributes to humoral hypercalcaemia of malignancy caused by LSCC [5]. These observations indicate the presence of some not-yet-discovered connections between both diseases besides the existing genetic relations.

To understand the molecular association between hyperkalemia and LSCC, we performed a large scale disease-gene relation data analysis and pathways analysis to explore potential pathways and networks that influencing both disorders. Then a mega-analysis was conducted to test the hypothesis that genes increase their activities with hyperkalemia may also play a role in the development of LSCC. Our study provides new insights into the understanding of the hyperkalemia-LSCC association and suggests a novel approach to examine influential genes for LSCC.

## Methods

### Large-scale literature data mining

Relation data for both hyperkalemia and LSCC were extracted from existing literature and analyzed using Pathway Studio (www.pathwaystudio.com) and then were downloaded into a genetic database Hyperkalemia_LSCC, hosted at http://database.gousinfo.com. The downloadable format of the database in excel is available at http://gousinfo.com/database/Data_Genetic/Hyperkalemia_LSCC.xlsx. Besides the list of analyzed genes (Hyperkalemia_LSCC→Hyperkalemia_genes, LSCC_genes, and Hyperkalemia_specific genes), supporting references for each disease-gene relation are presented at Hyperkalemia_LSCC→Hyperkalemia_gene_relation and LSCC_gene_relation, including titles of the references and the sentences describing identified disease-gene relationships. The information could be used to locate a detailed description of an association of a candidate gene with hyperkalemia and/or LSCC.

### Functional pathway analysis

After the hyperkalemia and LSCC relation data being curated, a literature-based functional pathway analysis was conducted with an aim to identify pathways/networks that linking both disorders. Moreover, after mega-analysis, pathway analysis was used to identify the potential biological linkage between LSCC and novel target genes from mega-analysis results. The pathway analysis was performed using the ‘Shortest Path’ module of Pathway Studio (www.pathwaystudio.com). A follow-up mega-analysis was used to validate the activity of the genes within the functional pathway.

### LSCC RNA expression datasets selection for mega-analysis

A keyword ‘lung squamous cell carcinomas’ was used to search LSCC expression datasets at the website of GEO (https://www.ncbi.nlm.nih.gov/geo/). Then, the datasets were further filtered by the following criteria: 1) The entry type is series; 2) The study type is RNA expression; 3) The sample size is no less than 10; 4) Sample organism is *Homo sapiens*; and 4) the studies were performed according to a case vs. normal control design.

To note, the selection of the data covers all LSCC expression array datasets from GEO, which is owned by the National Institutes of Health (NIH of USA). The datasets are publicly available, and no permission or confirmation is needed from any independent investigators. Moreover, datasets extraction has no selection bias in terms of publication journals, owner affiliations, and authors. In addition, the original data rather than the processed results of each dataset were used to perform the analysis in this study, which avoids possible noise caused by the independent data process.

### Mega-analysis models

To estimate the effect size of hyperkalemia related genes in the case of LSCC, we applied two models for the mega-analysis, including the fixed-effect model and random-effects model [6]. Results from both models were reported and compared. During the processing of the datasets, all the expression data were normalized and log2-transformed if not done in the original dataset.

The heterogeneity of the mega-analysis was analyzed to study the variance within and between different studies. In the case that the total variance Q is equal to or smaller than the expected between-study variance df, the eq. (1) will be set as 0, and a fixed-effect model was selected for the mega-analysis. Otherwise, a random-effects model was selected.

1 $$ ISq=\frac{Q- df}{Q}\ast 100\% $$

For each gene within an LSCC-expression dataset, the log fold change (LFC) was calculated and used as the index of effect size in mega-analysis. The Q-p represents the probability that the total variance is coming from within-study only. All analysis was conducted by an independently-developed MATLAB (R2017a) mega-analysis package.

The significant genes identified and selected from mega-analysis must be qualified by the following criteria: *p* < 0.005 and effect size abs (LFC) > 1. The details of the mega-analysis results were presented in the Hyperkalemia_LSCC→Mega-analysis.

### Multiple linear regression analysis for the risk factors on LSCC

A multiple linear regression analysis was utilized to study the possible influence of three factors on the gene expression change in LSCC: sample size, population region, and study age. *P*-values and 95% confidence interval (CI) were reported for each of the factors. The analysis was done in Matlab (R 2017a) with the ‘regress’ statistical analysis package.

## Results

### Disease-related genes from the literature database

**Fig. 1:**
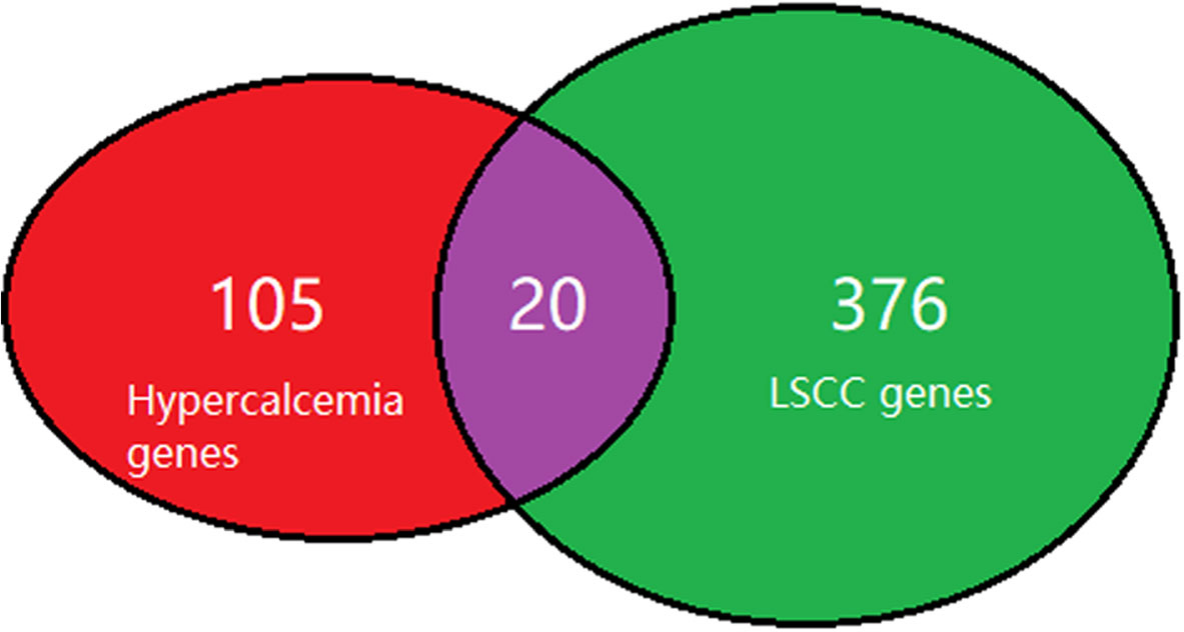
Venn diagram of the genes implicated with hyperkalemia and lung squamous cell carcinoma

We acquired both diseases related genes by using the Pathway Studio guided literature data-mining (www.pathwaystudio.com), yielding 125 Hyperkalemia-genes and 397 LSCC-genes (see Hyperkalemia_LSCC: hyperkalemia_genes and LSCC_genes). There was a significant overlap between the two gene groups (20 genes; *p*-value = 4.98e-15), with 105 out of the 125 hyperkalemia-related genes have not been implicated in LSCC (see Fig. 1).

### Common targets and regulators of hyperkalemia and LSCC

**Fig. 2:**
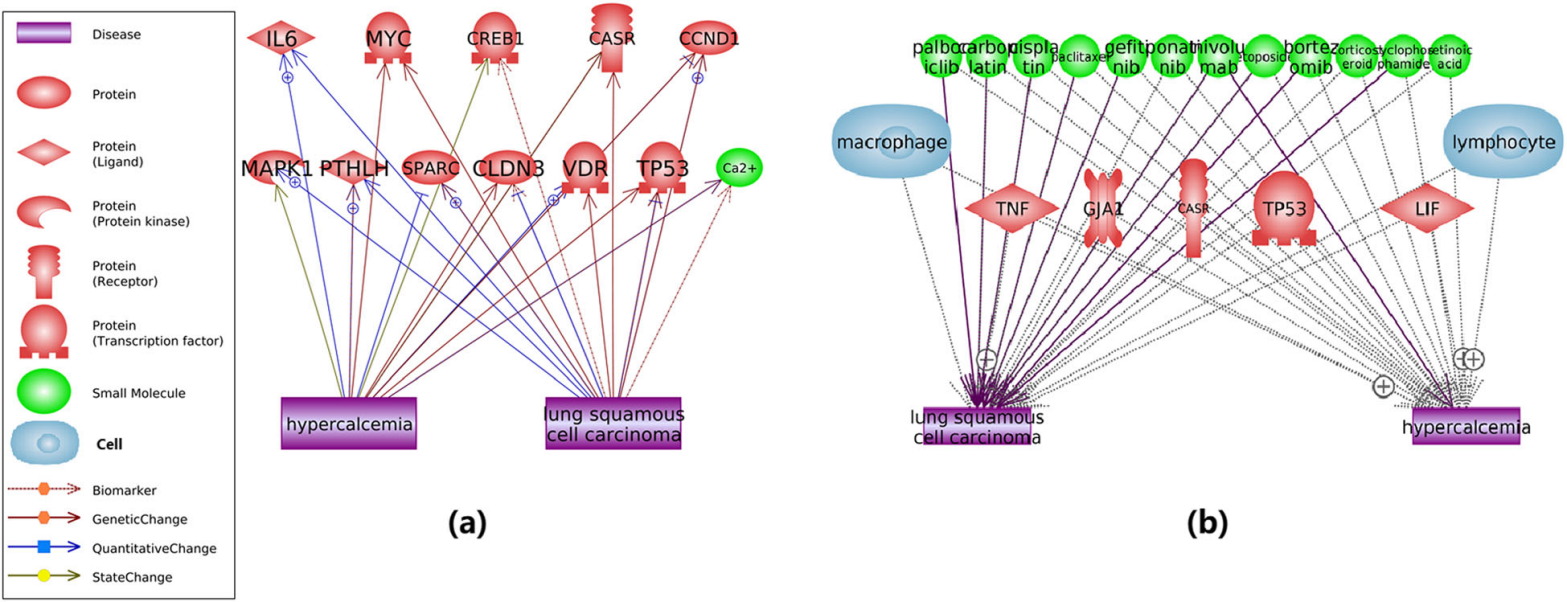
Common regulators and targets of hyperkalemia and lung squamous cell carcinoma. **a** Common targets; **b** Common regulators

Pathway analysis identified a common-target network (Fig. 2a) and a common-regulator network (Fig. 2b). On the one hand, we see that hyperkalemia and LSCC both regulating the activity of 11 proteins and one molecule (Ca2+). On the other hand, we identified 11 small molecules, 4 genes and 2 cell types as common regulators for both LSCC and hyperkalemia. These networks indicate that LSCC and hyperkalemia share a deep molecular basis in their pathological development.

### An interactive network connecting hyperkalemia and LSCC

**Fig. 3:**
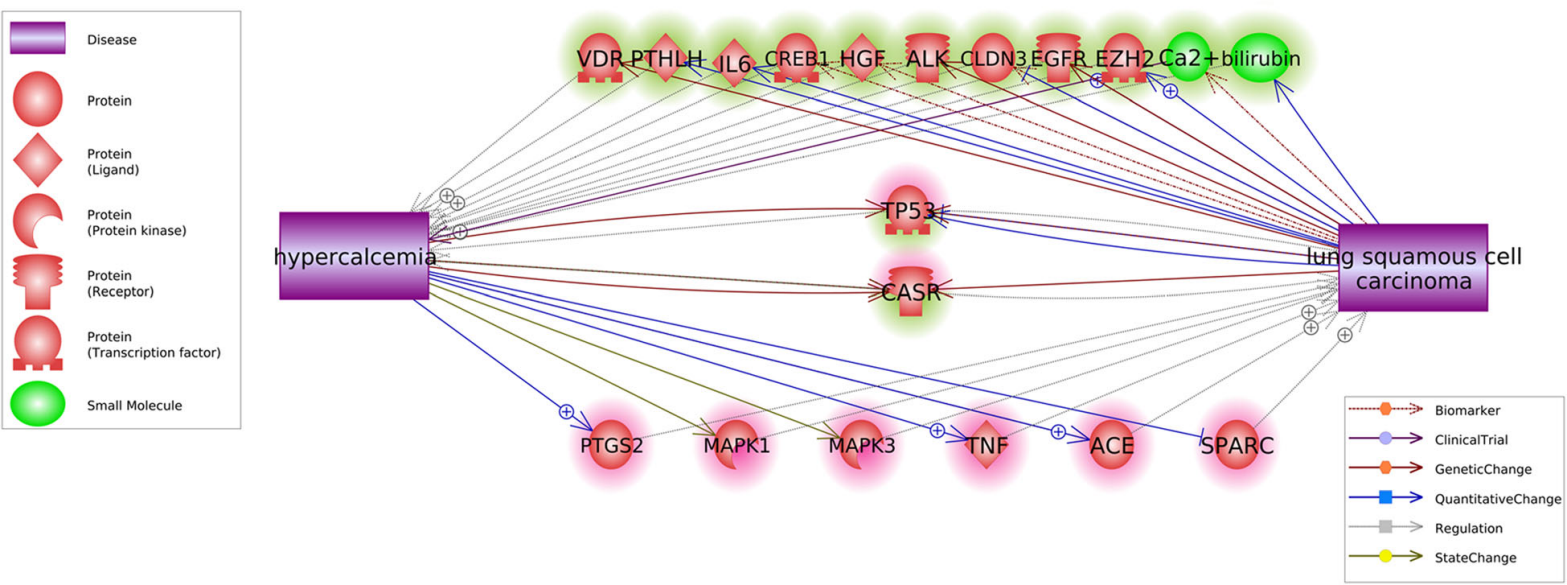
Interactive network through which hyperkalemia and lung squamous cell carcinoma may influence each other

Besides the common targets and common regulators, we also discovered an interactive network that connecting hyperkalemia and LSCC, as shown in Fig. 3. Interestingly, we see that not only LSCC could be a causal factor for hyperkalemia by regulating multiple molecules (Fig. 3, the molecules highlighted in green), but hyperkalemia also contributes to the development of LSCC through the influence on multiple genes (Fig. 3, the genes highlighted in red).

### Mega-analysis results

As shown in Table 1, a total of 11 independent RNA expression datasets qualified the filter criteria were utilized for the mega-analysis. The expression data acquired from 358 LSCC cases and 278 healthy controls were distributed in seven different countries with the study age ranged from 2 to 13 years.

**Table 1:** Datasets selection for mega-analysis

Study Name	Dataset GEOID	nControl	nCase	Country	Study Age
Nazarov et al.,2017	GSE84784	9	9	Luxembourg	2
Tong et al.,2016	GSE67061	8	69	China	3
Rousseaux et al.,2014	GSE30219	14	61	France	5
Mascaux et al.,2014	GSE33479	27	14	USA	5
Girard et al.,2012	GSE32036	59	12	USA	7
Philipsen et al.,2010	GSE19188	65	27	Netherlands	9
Takahashi et al.,2009	GSE11969	5	35	Japan	10
Boelens et al.,2009	GSE12472	28	35	Netherlands	10
Ishikawa et al.,2009	GSE2088	30	48	Japan	10
Boelens et al.,2008	GSE12428	28	34	Netherlands	11
Rosskopf et al.,2006	GSE6044	5	14	Germany	13

During the study, a log fold change (LFC) was estimated for each of the 105 hyperkalemia-specific genes from the majority of the 11 studies. Results showed that one gene, *SPP1*, presented significant increased LFC in the case of LSCC (*p* = 2.81e-6 and LFC = 2.64). Notably, *SPP1* was implicated as a hyperkalemia promoter. Heterogeneity analysis results showed that there was a significant between-study variance for mega-analysis (*p*-value = 2.81e-6), and therefore a random-effects model was selected. The related statistics were presented in Table 2.

**Table 2:** *SPP1* mega-analysis and regression analysis results

Gene Name	***SPP1***
**Random-Effects Model**	
Datasets included	11
LFC	2.64
STD of LFC	0.58
*p*-value	2.81e-6
ISQ	45.86
*p*-value-Q	0.048
**Multiple linear regression analysis**	
Sample Size	0.64
Population Region	0.054
Study Age	0.62

**Fig. 4:**
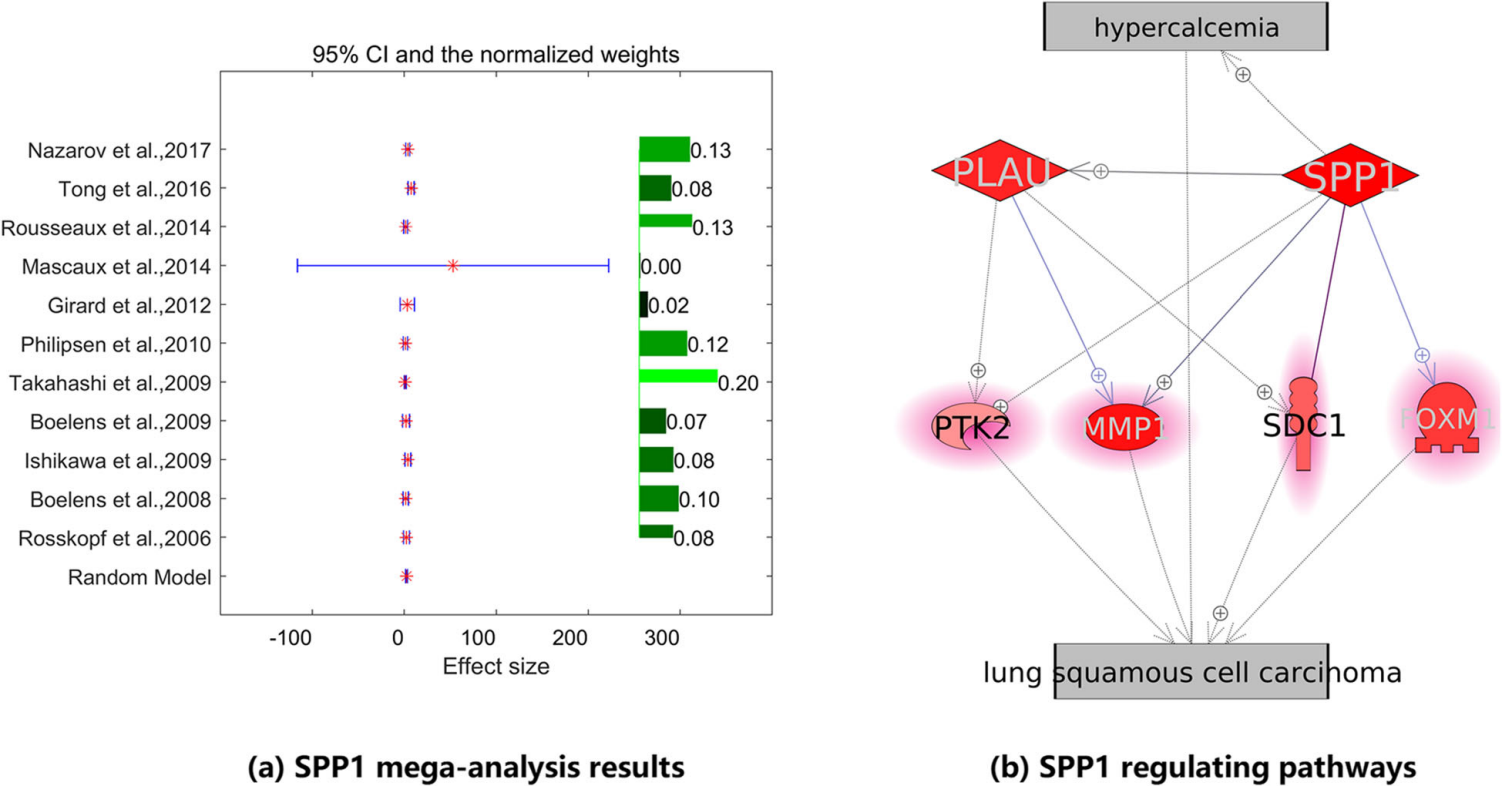
SPP1 presented significant effects in case of LSCC

Figure 4a presents the forest-plot from the mega-analysis for *SPP1*, including LFC, weight, and 95% confidence interval (CI). Figure 4b demonstrates the potential pathways through which *SPP1* may contribute to the pathologic development of LSCC. To note, the pathway in Fig. 4b was firstly built through literature-based pathway analysis, then we conducted another mega-analysis to test the activity of these molecules in case of LSCC, using the 11 LSCC RNA expression datasets listed in Table 1. Results show that *SPP1* activates the expression of multiple LSCC promoters, including *PTK2, MMP1, SDC1,* and *FOXM1,* both directly and through the activation of *PLAU* (Fig. 4b).

### MLR analysis results

Results from the MLR models showed that the population region (country) demonstrated significance for influencing the expression levels of *SPP1* (*p*-value = 0.054 and 0.036, respectively), as shown in Table 2. The sample size and study age were not significant risk factors.

## Discussion

Previous studies suggested the causal effects of cancers for hyperkalemia [2], as well as the negative influence of hyperkalemia on the prognosis of LSCC [3]. The purpose of our study is to explore the potential molecular mechanisms underlying the clinical association between hyperkalemia and LSCC. Based on the discovery, we made one further step to test the hypothesis that genes promoting hyperkalemia may also play roles in the development of LSCC.

Assisted by using the tool Pathway Studio (www.pathwaystudio.com), we conducted a large-scale literature data mining, covering over 40 million references to identify LSCC and hyperkalemia related genes, proteins, small molecules, and cell types. Based on the mining results, pathway analysis has been conducted to identify common targets and common regulators of LSCC and hyperkalemia. We also discovered a potential cross-network through which LSCC and hyperkalemia may connect with each other and mutually impact their pathologic development.

On the one hand, previous studies showed that LSCC and hyperkalemia influenced by multiple common molecules. For instance, macrophages play a vital role in the development of LSCC and hyperkalemia [7, 8]. Moreover, squamous carcinoma cell lines experiments showed that abnormal TP53 expression could cause hyperkalemia [9], while the mutation of TP53 has been suggested to play roles in the development of LSCC [10]. On the other hand, LSCC and hyperkalemia have an influence on multiple common molecules. It has been shown that increased calcium content is an indicator of LSCC [11], while hyperkalemia stimulates enzyme secretion and elevates calcium concentration [12]. It has also been suggested that the amplification and overexpression of MYC have been reported previously in both LSCC and hyperkalemia type carcinoma [13].

In addition, multiple evidence from previous studies indicated that hyperkalemia and LSCC could be mutual causal factors for each other. As shown in Fig. 3, besides the promotion of calcium [11], LSCC also activates multiple genes that produce hyperkalemia, including *EGFR*, *EZH2* [5, 14]. These studies partially explain the mechanism that LSCC induces hyperkalemia. On the other hand, hyperkalemia has been shown to stimulate the expression of multiple genes or proteins that promote the development of LSCC. For instance, hyperkalemia patients demonstrated elevated ACE levels [15], which lead to an increased risk of LSCC [16]. Hyperkalemia also stimulates the expression of PTGS2 [17], which plays an important role in the rat LSCC [18] and has been suggested as a therapeutic target for LSCC [19]. These studies indicated that hyperkalemia might be a risk factor that promotes the development of LSCC.

Based on the association study between LSCC and hyperkalemia, it is reasonable to hypothesize that genes promoting hyperkalemia may also play roles in the development of LSCC. Venn diagrams in Fig. 1 showed that 16% (20/125) genes implicated with hyperkalemia have also been suggested an association with LSCC. Fisher exact test showed that the probability that two random gene groups with the same size of genes (i.g., 125 and 397 genes) to have an overlap of 20 is less than 4.98e-15, which supports the hypothesis proposed here.

In addition, mega-analysis discovered one hyperkalemia-specific promoter, *SPP1,* also presented significantly increased expression in the case of LSCC (*p* = 2.81e-6; LFC = 2.64) (Table 2, Fig. 4b). MLR analysis results suggested that the population region (country) was a factor that affects the expression level of *SPP1* in the case of LSCC (*P*-value = 0.054). Pathway analysis showed that *SPP1* might promote the pathological development of LSCC through the stimulation of the expression of multiple genes, as shown in Fig. 4b. Overexpression of *SPP1* was reported to be associated with poor prognosis, progression, migration, and invasion of multiple cancers [20–23]. The elevated expression of *SPP1* could result in the up-regulation of *SDC1* signaling (gene colored in red, Fig. 2), which promotes the metastasis in LSCC [24]. Moreover, *SPP1* induces increased *PLAU* production that indirectly activates *MMP1,* which promotes tumor progression and LSCC cell migration and invasion [25, 26]. The pathway presented in Fig. 4b provides further support for the hypothesis that genes promoting hyperkalemia may also play roles in the development of LSCC. To note, the activities of the genes within the *SPP1* signaling pathway (Fig. 4b) have been validated by the mega-analysis using the 11 independent datasets listed in Table 1.

To note, this study was designed to explore the mechanism of LSCC-hyperkalemia mechanism at the molecular level. The shared genetic pathways and molecular targets and regulators may partially explain the mutual influence of LSCC and hyperkalemia. However, our results did not suggest a causal effect of hyperkalemia on LSCC.

## Conclusion

Our results suggested that LSCC and hyperkalemia have a shared pathological basis and are mutual influential factors for each other. Genes associated with hyperkalemia may also play roles for the development of LSCC, and *SPP1* could be a promoter for both hyperkalemia and LSCC.

## Data Availability

All data supporting the findings of this study are available from the corresponding author in response to a reasonable request.

## Abbreviations

LSCC: Lung squamous cell carcinomas
LFC: Log fold change
CI: Confidence interval
